# Alzheimer’s Disease Concurrent With Patent Foramen Ovale-Related Lacunar Infarcts and Extensive White Matter Hyperintensities: A Case Highlighting Biomarker-Clinical Staging Discordance

**DOI:** 10.7759/cureus.93738

**Published:** 2025-10-02

**Authors:** Jieli Geng, Chenpeng Zhang, Nan Zhi, Xingchen Dai, Gang Wang

**Affiliations:** 1 Department of Neurology, Renji Hospital, Shanghai Jiao Tong University School of Medicine, Shanghai, CHN; 2 Department of Nuclear Medicine, Renji Hospital, Shanghai Jiao Tong University School of Medicine, Shanghai, CHN

**Keywords:** alzheimer’s disease, biomarker staging, lacunar infarction, patent foramen ovale, white matter hyperintensities

## Abstract

An elderly man with progressive memory decline met the 2024 National Institute on Aging and the Alzheimer's Association (NIA-AA) biological criteria for Alzheimer’s disease (AD) - classified as stage C biologically but mild dementia clinically - revealing a clear staging mismatch. Recognizing this discordance led us to investigate additional comorbidities. He was found to have extensive white matter hyperintensities (WMHs) (Fazekas grade 3) and experienced multiple recurrent lacunar infarcts. Subsequent evaluation identified a patent foramen ovale (PFO) causing right-to-left shunting and paradoxical embolism. This case underscores the necessity of detecting biological-clinical discordance in AD, actively screening for cerebrovascular comorbidities such as WMHs and infarcts, and accurately identifying underlying causes to optimize patient management and enrich AD clinical research.

## Introduction

In recent years, Alzheimer’s disease (AD) has entered the era of disease-modifying therapy (DMT), supported by Phase III trials of anti-amyloid-beta (Aβ) monoclonal antibodies such as lecanemab [1] and donanemab [2], and these therapies are now available in China [3]. Meanwhile, amyloid PET imaging is increasingly utilized in Chinese tertiary memory clinics, significantly enhancing diagnostic accuracy and guiding treatment decisions [4]. Against this backdrop, patient education and clinical messaging have focused heavily on AD pathology and targeted therapies. However, this singular focus on AD itself may result in underappreciation of comorbid conditions - particularly cerebrovascular contributors to cognitive impairment. Vascular comorbidities are common in AD and contribute to the clinical heterogeneity of dementia. In a community-based neuropathological study, 38% of individuals with dementia had both AD and cerebral infarcts, highlighting the high prevalence of mixed pathologies in older adults [5].

According to the 2024 National Institute on Aging and the Alzheimer's Association (NIA-AA) criteria [6], AD can be diagnosed based on abnormal biomarkers such as amyloid PET. Biological staging is divided into stages A-D based on amyloid and tau PET patterns, while clinical staging ranges from stage 1 (preclinical) to stage 6 (severe dementia). The integrated biological-clinical staging matrix maps these dimensions together, with the typical trajectory progressing diagonally from stage 1A to 4-6D. For example, patients with intermediate biological stage C are generally expected to present at clinical stage 3 (i.e., 3C). However, considerable individual variability exists: patients with worse clinical staging than expected (e.g., 4-6C) often have additional comorbid pathology, while those with milder symptoms than expected may have higher cognitive reserve.

This report presents a patient who met the updated 2024 NIA-AA biological criteria for AD (A+T+) [6]. However, the severity of cognitive impairment in this case was significantly greater than expected, prompting further evaluation into potential comorbidities. Detailed clinical investigation revealed recurrent lacunar infarctions likely related to a patent foramen ovale (PFO), as well as extensive periventricular white matter hyperintensities (WMHs), graded Fazekas 3, and accompanied by cerebral microbleeds. These findings suggest a multifactorial etiology contributing to the patient's cognitive decline.

This case provides a compelling example that, despite the increasing focus on DMTs and biomarker-defined AD, comorbid conditions must not be overlooked. A thorough assessment of mixed etiologies is essential not only for accurate diagnosis and personalized care but also for ensuring the scientific rigor of real-world DMT research. Identifying and addressing comorbidities may be critical to optimizing therapeutic outcomes and reducing misclassification in the DMT era.

## Case presentation

A 77-year-old man with 12 years of formal education presented with a three-year history of progressive memory decline, predominantly affecting recent memory, accompanied by mild disorientation. He denied language difficulties. His history included hypertension managed with regular medication.

Neurological examination was unremarkable: normal muscle strength and tone, symmetrical tendon reflexes, and negative Babinski signs. Neuropsychological evaluation showed a Montreal Cognitive Assessment (MoCA) score of 14/30, with preserved daily functioning (as evidenced by an Instrumental Activities of Daily Living Scale (IADL) score of 14 and a Physical Self-Maintenance Scale (PSMS) score of 6) and a Hamilton Depression Rating Scale (HAMD) score of 4, indicating early mild dementia without depressive symptoms [7-10]. While the patient retained full activities of daily living (ADL) scores, his family reported subtle but functionally relevant impairments, such as forgetting whether clothes had already been hung out to dry or repeating simple household tasks. He also experienced occasional navigational difficulties in less familiar environments, though he remained oriented in familiar settings. No expressive or receptive language deficits were observed. Routine laboratory investigations encompassing hematological, metabolic (including hepatic, renal, lipid, and glucose panels), endocrine, infectious, nutritional, and oncological parameters were largely unremarkable, except for a mild normocytic anemia. These tests provided an initial screen for metabolic, paraneoplastic, and infectious etiologies. The relevant results are summarized in Table 1.

**Table 1:** Summary of comprehensive laboratory workup All values were within normal reference ranges, except for mild normocytic anemia. WBC: White blood cell count; RBC: Red blood cell count; PLT: Platelet count; ALT: Alanine transaminase; AST: Aspartate transaminase; HDL: High-density lipoprotein; LDL: Low-density lipoprotein; K⁺: Potassium; Na⁺: Sodium; Cl⁻: Chloride; CLIA: Chemiluminescence immunoassay; AFP: Alpha-fetoprotein; CEA: Carcinoembryonic antigen; CA: Carbohydrate antigen; HbA1c: Glycated hemoglobin

Test	Result	Reference Range	Unit
WBC	6.87	3.5-9.5	×10⁹/L
RBC	3.38	4.3-5.8	×10¹²/L
Hemoglobin	113	130-175	g/L
PLT	151	125-350	×10⁹/L
ALT	12.1	9-50	U/L
AST	11	15-40	U/L
Creatinine	73	57-111	μmol/L
Uric acid	402	155-428	μmol/L
Fasting blood glucose	6.04	3.9-6.1	mmol/L
HbA1c	5.7	4-6	%
Total cholesterol	3.86	Desirable: <5.20	mmol/L
Triglycerides	1.17	Desirable: <1.70	mmol/L
HDL-cholesterol	1.87	0.90-2.00	mmol/L
LDL-cholesterol	1.37	Varies by risk	mmol/L
Lipoprotein(a)	232	0.0-300.0	mg/L
Albumin	41.6	40-55	g/L
K⁺	3.75	3.5-5.3	mmol/L
Na⁺	143.5	137-147	mmol/L
Cl⁻	106.1	99-110	mmol/L
Homocysteine	9.3	3.0-17.0	μmol/L
Folic acid (folate)	5.4	3.1-19.9	μg/L
Vitamin B12	582	180-914	pg/mL
Treponemal antibody (CLIA)	Negative	Negative	-
HIV antibody	Negative	Negative	-
AFP	2.24	0-7	ng/mL
CEA	4.68	0-4.7	ng/mL
CA19-9	21.7	0-27	U/mL
CA50	10.5	0-25	IU/mL
CA242	5.24	0-10	U/mL
CA125	10.1	0-35	U/mL

**Figure 1:**
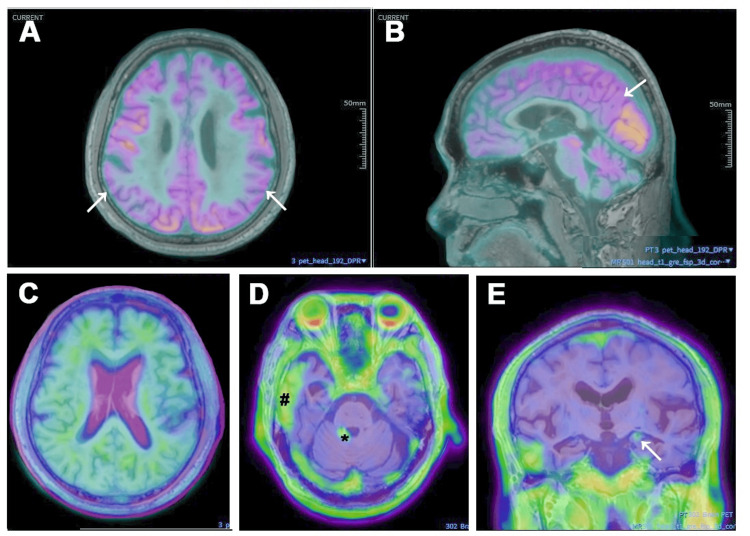
AD-related multimodal PET imaging (A-B) FDG PET demonstrates hypometabolism in bilateral precuneus and temporoparietal regions (arrows). (C) Amyloid PET (AV45) reveals widespread cortical Aβ deposition, consistent with AD pathology. (D-E) Tau PET highlights tau accumulation in the hippocampi (arrow), lateral temporal lobes (#), and cerebellum (*), consistent with AD pathology. AD: Alzheimer’s disease; FDG: Fluorodeoxyglucose; AV45: Florbetapir; Aβ: Amyloid-beta

**Figure 2:**
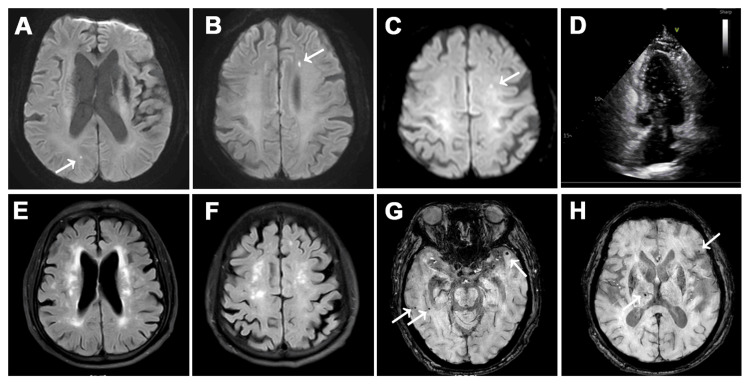
Follow-up MRI and right heart contrast echocardiography (A-C) Brain MRI (DWI) shows acute lacunar infarcts in the left centrum semiovale (A) (October 8, 2024) and a new infarct in the right occipital (B) and left frontal lobes (C) (February 18, 2025) (arrows). (D) Right heart contrast echocardiography reveals immediate entry of more than 30 microbubbles into the left atrium and ventricle following right-sided chamber filling, indicating a large right-to-left shunt consistent with PFO. (E-F) MRI FLAIR depicts extensive periventricular (E) and subcortical (F) white matter hyperintensities (Fazekas grade 3). (G-H) MRI SWI displays multiple cerebral microbleeds in the basal ganglia and cortex (arrows). DWI: Diffusion-weighted imaging; PFO: Patent foramen ovale; FLAIR: Fluid-attenuated inversion recovery; SWI: Susceptibility-weighted imaging

Fluorine-18-labeled fluorodeoxyglucose (^18^F-FDG) PET demonstrated hypometabolism in the bilateral precuneus and temporoparietal regions (Figures 1A-1B). Florbetapir (AV45) amyloid PET revealed widespread cortical Aβ deposition (Figure 1C), with a global standardized uptake value ratio (SUVR) of 1.26 (cutoff: 1.11), supporting a positive scan consistent with AD pathology. Tau PET showed tau accumulation in the hippocampi, lateral temporal lobes, and cerebellum, as indicated in Figures 1D-1E. MRI revealed an acute infarct in the left centrum semiovale (October 8, 2024), and follow-up diffusion-weighted imaging (DWI) on February 18, 2025, showed new lacunar infarcts in the right occipital and left frontal lobes - consistent with recurrent subcortical infarctions - as depicted in Figures 2A-2C. Additionally, fluid-attenuated inversion recovery (FLAIR) images demonstrated extensive periventricular and subcortical WMHs, graded Fazekas 3 (Figures 2E-2F), and susceptibility-weighted imaging (SWI) sequences showed multiple cerebral microbleeds in both basal ganglia and cortical regions (Figures 2G-2H). These imaging findings corroborated a high burden of cerebral small vessel disease.

Initial therapy included donepezil for cognitive symptoms and clopidogrel plus atorvastatin for secondary stroke prevention, alongside continued blood pressure control.

During follow-up, he had several transient speech disturbances that resolved spontaneously. Repeat MRI showed new lacunar infarcts in the right occipital and left frontal lobes (as described above). A comprehensive stroke workup showed no significant vascular stenosis or atrial fibrillation. Contrast-enhanced transthoracic echocardiography with bubble study confirmed a large right-to-left shunt, compatible with PFO. Transesophageal echocardiography was not performed due to procedural risk related to the patient’s age, and transcatheter closure was not pursued. Anticoagulation with rivaroxaban 20 mg daily was initiated. No new infarcts were detected during the following three months of observation.

## Discussion

Despite meeting the 2024 NIA-AA biological criteria for AD (A+T+), the patient exhibited cognitive and functional impairments greater than expected for his biological stage [6]. Tau-PET showed deposition in neocortical regions consistent with biological stage C. Clinically, he presented with early mild dementia (clinical stage 4), which is more advanced than typically expected for biological stage C. This staging mismatch prompted further investigation into comorbidities. While standardized ADL tools showed preserved daily functioning, caregiver-reported behaviors such as repeated tasks and subtle topographical difficulties suggested early but functionally meaningful decline. His MoCA score (14/30) also supported a clinical diagnosis of mild dementia. Comprehensive assessment revealed multiple lacunar infarcts likely due to a PFO and extensive periventricular WMHs, graded as Fazekas 3, with cerebral microbleeds. These findings indicate a multifactorial rather than pure AD etiology for the patient’s cognitive decline.

AD often coexists with cerebrovascular pathology: up to 84% of elderly brains show vascular lesions alongside AD changes [11]. Such overlap typically worsens cognitive outcomes beyond what either pathology alone would predict. Ischemic stroke - especially when recurrent - markedly accelerates decline, with approximately 30-60% of survivors experiencing new cognitive impairment within a year [12]. Lacunar strokes notably impair processing speed, executive function, and motor dexterity [13]. In our patient, recurrent subcortical lacunar infarcts accompanied early AD pathology and led to multidomain cognitive decline, consistent with a mixed vascular-AD impairment pattern. Therefore, identifying causes of recurrent infarcts and implementing effective secondary prevention is crucial to prevent further cognitive deterioration.

The patient also displayed extensive subcortical and periventricular WMHs, rated Fazekas grade 3, accompanied by multiple microbleeds in the basal ganglia and cortex. The Fazekas scale is a widely used visual rating tool for WMH severity in clinical and research settings, with grade 3 indicating severe, confluent lesions [14]. WMHs are common MRI findings in older adults [15] and are known to impair processing speed and executive function [16]. Clinically, the patient exhibited slowed responses, difficulties with daily tasks, and measurable executive dysfunction - symptoms corresponding to a high WMH burden. These findings may underlie the patient's impaired executive function and slowed processing, as reflected in MoCA subdomain performance and caregiver reports.

Although WMHs are traditionally considered imaging markers of vascular cognitive impairment, their etiology is heterogeneous [17]. In Alzheimer’s cohorts, WMH volume has been linked to amyloid pathology, suggesting a subset of WMHs may arise from neurodegenerative rather than purely vascular mechanisms [18,19]. Additionally, PFO has been linked to “silent” WMHs, possibly due to microembolism [20]. These findings underscore the complex interplay between vascular and AD-related mechanisms in WMH pathogenesis, reinforcing the importance of considering mixed etiologies when evaluating cognitive impairment.

By applying the integrated staging from the 2024 NIA-AA criteria, this case identified multiple comorbidities coexisting with AD that may have exacerbated cognitive decline. In clinical practice, a comprehensive evaluation of AD patients is essential for accurate diagnosis, personalized management, and ensuring methodological rigor in real-world DMT studies.

## Conclusions

This case illustrates the importance of recognizing biological-clinical discordance in AD and considering vascular comorbidities such as WMHs and lacunar infarctions during diagnostic workup. Such an approach may improve diagnostic accuracy, inform individualized treatment decisions, and support more rigorous real-world evaluation of DMTs.

## References

[1] van Dyck CH, Swanson CJ, Aisen P (2023). Lecanemab in early Alzheimer's disease. N Engl J Med.

[2] Sims JR, Zimmer JA, Evans CD (2023). Donanemab in early symptomatic Alzheimer disease: the TRAILBLAZER-ALZ 2 randomized clinical trial. JAMA.

[3] Zhi N, Ren R, Qi J (2025). The China Alzheimer report 2025. Gen Psychiatr.

[4] Chen KL, Wang MY, Wu J (2024). Incremental value of amyloid PET in a tertiary memory clinic setting in China. Alzheimers Dement.

[5] Schneider JA, Arvanitakis Z, Bang W, Bennett DA (2007). Mixed brain pathologies account for most dementia cases in community-dwelling older persons. Neurology.

[6] Jack CR Jr, Andrews JS, Beach TG (2024). Revised criteria for diagnosis and staging of Alzheimer's disease: Alzheimer's Association Workgroup. Alzheimers Dement.

[7] Nasreddine ZS, Phillips NA, Bédirian V (2005). The Montreal Cognitive Assessment, MoCA: a brief screening tool for mild cognitive impairment. J Am Geriatr Soc.

[8] Lawton MP, Brody EM (1969). Assessment of older people: self-maintaining and instrumental activities of daily living. Gerontologist.

[9] Katz S, Ford AB, Moskowitz RW, Jackson BA, Jaffe MW (1963). Studies of illness in the aged. The index of ADL: a standardized measure of biological and psychosocial function. JAMA.

[10] Hamilton M (1960). A rating scale for depression. J Neurol Neurosurg Psychiatry.

[11] Attems J, Jellinger KA (2014). The overlap between vascular disease and Alzheimer's disease - lessons from pathology. BMC Med.

[12] El Husseini N, Katzan IL, Rost NS (2023). Cognitive impairment after ischemic and hemorrhagic stroke: a scientific statement from the American Heart Association/American Stroke Association. Stroke.

[13] Jacova C, Pearce LA, Costello R, McClure LA, Holliday SL, Hart RG, Benavente OR (2012). Cognitive impairment in lacunar strokes: the SPS3 trial. Ann Neurol.

[14] Fazekas F, Chawluk JB, Alavi A, Hurtig HI, Zimmerman RA (1987). MR signal abnormalities at 1.5 T in Alzheimer's dementia and normal aging. AJR Am J Roentgenol.

[15] Duering M, Biessels GJ, Brodtmann A (2023). Neuroimaging standards for research into small vessel disease - advances since 2013. Lancet Neurol.

[16] Prins ND, Scheltens P (2015). White matter hyperintensities, cognitive impairment and dementia: an update. Nat Rev Neurol.

[17] Wang J, Zhou Y, He Y (2022). Impact of different white matter hyperintensities patterns on cognition: a cross-sectional and longitudinal study. Neuroimage Clin.

[18] Garnier-Crussard A, Cotton F, Krolak-Salmon P, Chételat G (2023). White matter hyperintensities in Alzheimer's disease: beyond vascular contribution. Alzheimers Dement.

[19] Shirzadi Z, Schultz SA, Yau WW (2023). Etiology of white matter hyperintensities in autosomal dominant and sporadic Alzheimer disease. JAMA Neurol.

[20] Badea RȘ, Mihăilă-Bâldea S, Ribigan A (2024). PFO-spectrum disorder: two different cerebrovascular diseases in patients with PFO as detected by AI brain imaging software. Front Neurol.

